# A CCTA coronary plaque radiomic model for predicting major adverse cardiovascular events in patients with coronary artery disease

**DOI:** 10.3389/fcvm.2026.1886018

**Published:** 2026-08-20

**Authors:** Yue Zhang, Hongkun Zhang, Minglang Yang, Jingyi Wang, Jing Wen, Baoying Zhao, Xu Dai

**Affiliations:** 1 The First Clinical College, Liaoning University of Traditional Chinese Medicine, Shenyang, Liaoning, China; 2 Clinical Science, Philips (China) Investment Co., Ltd., Shenyang, Liaoning, China; 3 Department of Radiology, The Affiliated Hospital of Liaoning University of Traditional Chinese Medicine, Shenyang, Liaoning, China

**Keywords:** coronary artery disease, coronary computed tomography angiography, coronary plaque, major adverse cardiovascular events, radiomics

## Abstract

**Introduction:**

Coronary artery disease (CAD) remains the leading cause of cardiovascular mortality worldwide, and early accurate risk stratification for major adverse cardiovascular events (MACE) is critical for improving clinical outcomes. Coronary computed tomography angiography (CCTA) is the cornerstone of non-invasive plaque assessment, yet conventional morphological evaluation fails to capture plaque microheterogeneity, limiting the accuracy of traditional risk prediction. We developed and validated a multidimensional model integrating clinical, plaque morphological, and CCTA-derived radiomic features to predict MACE, and quantified the incremental prognostic value of radiomics.

**Methods:**

A total of 567 patients with CCTA-confirmed CAD were retrospectively enrolled and randomly split 7:3 into training (*n* = 398) and test (*n* = 169) sets. From 1,833 extracted radiomic features, hierarchical dimensionality reduction selected core features to construct a radiomic score. Four models were established: clinical + plaque, clinical + radiomics, plaque + radiomics, and all features. Model performance was assessed using area under the receiver operating characteristic curve (AUC), calibration curves, and decision curve analysis. Incremental value was evaluated via the DeLong test, integrated discrimination improvement (IDI), and net reclassification improvement (NRI). Model interpretability was elucidated via SHapley Additive exPlanations (SHAP).

**Results and Discussion:**

In the test set, the full model achieved an AUC of 0.840 (95% CI: 0.774–0.907), accuracy of 0.822, and Brier score of 0.150, significantly outperforming the clinical + plaque model (AUC = 0.771, *P* = 0.016). The full model yielded an IDI of 0.203 (95% CI: 0.151–0.256), categorical NRI of 0.379 (95% CI: 0.245–0.508), and continuous NRI of 1.151 (95% CI: 0.88–1.416). SHAP analysis identified texture features reflecting plaque grayscale heterogeneity as the primary drivers of MACE prediction. These findings demonstrate that combining clinical, plaque, and radiomic features significantly improves MACE prediction in CAD, and that radiomics offers substantial incremental prognostic value, providing a reliable non-invasive risk stratification tool.

## 1 Introduction

Coronary artery disease (CAD) remains the leading cause of cardiovascular morbidity and mortality worldwide, imposing a substantial and growing burden on global public health systems (1). Despite remarkable advances in percutaneous coronary intervention (PCI) and pharmacotherapy, the incidence and mortality rates of acute coronary syndrome (ACS) remain unacceptably high (2). Accumulating evidence indicates that approximately 70% of acute cardiovascular events do not originate from severely stenotic lesions but are instead triggered by the sudden rupture or erosion of non-obstructive vulnerable plaques (3). Consequently, the accurate identification of asymptomatic or stable CAD patients harboring high-risk plaques prior to the occurrence of adverse events, thereby enabling early intervention and individualized management, represents a pivotal strategy for reducing long-term adverse outcomes and curbing the rising trend of cardiovascular mortality (4, 5).

Coronary computed tomography angiography (CCTA) has been recommended as the first-line non-invasive imaging modality for patients with suspected CAD, owing to its non-invasive nature, high spatial resolution, and capacity for three-dimensional reconstruction of the coronary tree (6). CCTA not only permits accurate quantification of luminal stenosis severity but also enables detailed visualization of plaque morphological characteristics, including traditional high-risk plaque markers such as positive remodeling, low-attenuation plaque, spotty calcification, and the napkin-ring sign (7, 8). Several large-scale prospective clinical trials, including SCOT-HEART and PROMISE, have confirmed that these traditional high-risk plaque features are independent predictors of major adverse cardiovascular events (MACE). However, a growing body of clinical studies and meta-analyses has demonstrated that reliance on conventional visually assessed high-risk plaque features yields a positive predictive value for MACE of merely 10%–20%, substantially limiting their clinical utility (9). The fundamental reason for this limitation is that traditional qualitative and quantitative assessments capture only the macroscopic morphological alterations of plaques and fail to reveal the complex microstructural heterogeneity and biological activity of atherosclerotic lesions, which constitute the essential determinants of plaque vulnerability and rupture risk (9).

In recent years, radiomics has emerged as a novel quantitative image analysis technology that offers a promising pathway for addressing this clinical challenge. Radiomics enables the high-throughput extraction of vast numbers of quantitative features from medical images that are imperceptible to the unaided human eye, transforming visual images into mineable high-dimensional data and thereby revealing the underlying pathophysiological information of lesions (10, 11). Existing studies have demonstrated that CCTA-based plaque radiomics holds considerable potential for discriminating vulnerable plaques, predicting hemodynamically significant stenosis, and assessing perivascular inflammation (12–14). However, whether radiomics can provide independent and significant incremental predictive value for MACE risk stratification beyond the integration of traditional clinical risk factors and macroscopic plaque characteristics remains inadequately validated through large-sample, multidimensional systematic investigations (15). The majority of existing models focus predominantly on imaging features in isolation, often neglecting the synergistic prognostic value derived from integrating traditional clinical risk factors with macroscopic plaque characteristics (16).

Against this background, the present study aimed to extract radiomic features from target coronary plaques on CCTA images and integrate these features with clinical baseline data and conventional plaque morphological parameters to construct a multidimensional MACE risk prediction model. Through rigorous internal validation and clinical utility assessment, multiple statistical approaches—including the DeLong test, integrated discrimination improvement (IDI), and net reclassification improvement (NRI)—were employed to comprehensively quantify the incremental value of radiomics. Furthermore, SHapley Additive exPlanations (SHAP) analysis was introduced to enhance model interpretability. This study aims to develop objective, interpretable imaging biomarkers to enable non-invasive, individualized risk stratification for patients with CAD.

## 2 Materials and methods

### 2.1 Study population

**Figure 1:**
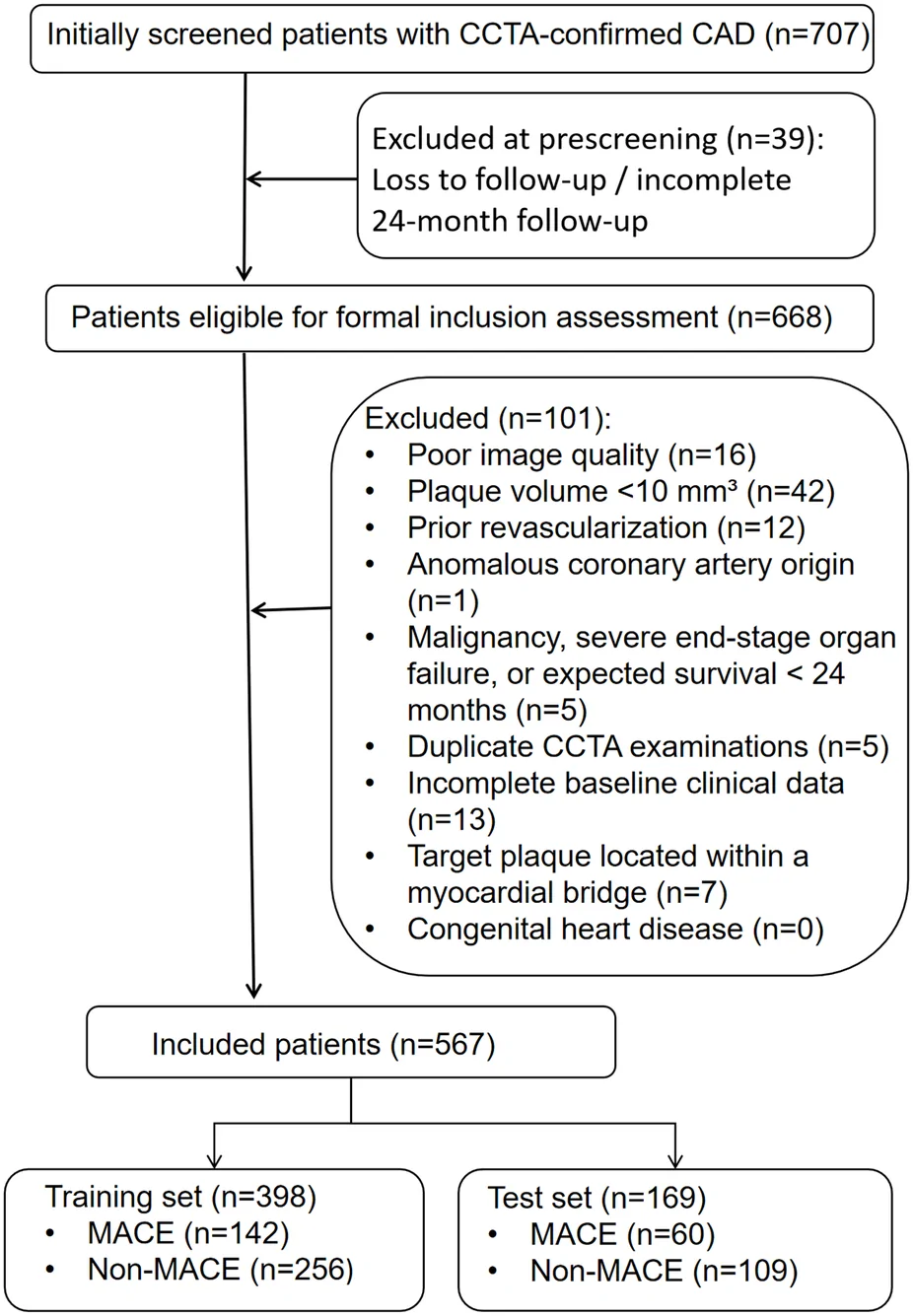
Patient inclusion and grouping flowchart. CAD, coronary artery disease; CCTA, coronary computed tomography angiography; MACE, major adverse cardiovascular events.

This retrospective study consecutively screened 707 patients with coronary artery disease (CAD) confirmed by CCTA at the Medical Imaging Center of the Affiliated Hospital of Liaoning University of Traditional Chinese Medicine between January 2020 and December 2023. After excluding patients with loss to follow-up or incomplete 24-month follow-up (*n* = 39), poor image quality (*n* = 16), plaque volume <10 mm^3^ (*n* = 42), prior coronary revascularization (*n* = 12), coronary anomalies (*n* = 1), malignancy, severe end-stage organ failure, or expected survival <24 months (*n* = 5), duplicate CCTA examinations (*n* = 5), incomplete baseline clinical data (*n* = 13), and target plaque located within a myocardial bridge (*n* = 7), a total of 567 patients were ultimately included. No patients were excluded for congenital heart disease. The patient screening and grouping process is illustrated in Figure 1.

The primary endpoint of this study was major adverse cardiovascular events (MACE), defined as a composite of cardiovascular death, non-fatal acute myocardial infarction, and ischemia-driven unplanned coronary revascularization (performed ≥6 weeks after the index CCTA examination). Secondary endpoints within the composite MACE definition included rehospitalization for unstable angina, new-onset congestive heart failure, and malignant arrhythmia. MACE occurrence was ascertained through hospital electronic medical records and telephone follow-up. The median follow-up duration was 24 months (IQR: 22.0–25.0 months), with a relatively concentrated follow-up period and limited temporal bias. None of the 567 patients ultimately included were lost to follow-up, ensuring complete and reliable follow-up data. For patients in the MACE group, follow-up time was defined as the interval from baseline CCTA to the first occurrence of any adverse event. Baseline clinical data, laboratory test results, and CCTA-derived plaque imaging parameters were collected for all patients.

This retrospective study was approved by the local Institutional Review Board and Ethics Committee [Approval No. Y2026012CS(KT)-012-01]. Given the retrospective, observational nature of the study, the exclusive use of anonymized data generated during routine clinical practice, the absence of any additional intervention, and the removal of all personally identifiable information, the Ethics Committee granted a waiver of informed consent. All data were anonymized and handled in accordance with the ethical principles of the Declaration of Helsinki.

### 2.2 Clinical data and CCTA acquisition

This study retrospectively collected clinical baseline characteristics, laboratory test results, and follow-up outcome data from the 567 eligible patients. Clinical data encompassed age, sex, smoking history, alcohol consumption history, and medical history of hypertension, diabetes mellitus, and hyperlipidemia. Laboratory parameters included total cholesterol, triglycerides, high-density lipoprotein cholesterol, low-density lipoprotein cholesterol, C-reactive protein, and fasting blood glucose.

All CCTA scans were performed using a 256-slice CT scanner (Brilliance iCT, Royal Philips) with prospective electrocardiographic gating. The scanner specifications included a collimator of 128 × 0.625 mm, a tube voltage of 100 or 120 kV, automatic tube current adjustment, a slice thickness of 0.9 mm, a slice increment of 0.625 mm, and a rotation time of 270 ms. The acquisition window was set at 30%–80% of the R-R interval. For optimal image quality, all patients received sublingual nitroglycerin to dilate the coronary arteries, and a β-blocker (metoprolol, 25–50 mg, orally) was administered as needed 30 min before the CCTA scan to achieve a target heart rate below 65 beats per minute. A non-ionic contrast medium (iopromide 350 mgI/mL, Hengrui Pharmaceutical Co., Ltd., Jiangsu, China) was administered via a high-pressure syringe at a flow rate of 5.0 mL/s through an antecubital vein, followed by a 40 mL saline flush at the same flow rate.

### 2.3 Conventional plaque assessment

In this study, plaque analysis was performed exclusively on coronary segments with a diameter > 1.5 mm. A uniform target plaque selection rule was applied to all enrolled patients, based solely on baseline CCTA images without reference to any follow-up outcome information. For each patient, the single plaque exhibiting the most severe luminal stenosis and the greatest plaque burden within the coronary artery was selected as the target plaque. If multiple plaques were present within the same vessel or across multiple vessels, the same criteria were applied to select a single target plaque. This selection rule was identical for both the MACE group and the non-MACE group. All plaque delineation was performed by radiologists who were completely blinded to patient follow-up outcomes and group assignment.

Quantitative measurements of target plaques were performed using the fully automated plaque analysis module of the Neusoft Vasis Coronary CTA, including remodeling index, stenosis severity, vulnerable plaque assessment, and plaque burden. Plaque burden was defined as plaque volume divided by vessel volume. Vulnerable plaque characteristics were evaluated for each lesion, including positive remodeling, low-attenuation plaque, spotty calcification, and the napkin-ring sign. High-risk plaque (HRP) was defined as a lesion exhibiting at least two of the aforementioned vulnerable plaque characteristics.

### 2.4 Radiomics feature extraction and selection

All CCTA images were acquired and stored in a unified DICOM format. Prior to uploading to the analysis software, image denoising and standardization preprocessing were performed. Plaque analysis was conducted using fixed window width and window level settings to ensure the stability and consistency of radiomic feature extraction. Regions of interest (ROIs) of coronary plaques were manually delineated slice-by-slice through consensus by two radiologists with over 5 years of experience in cardiovascular imaging diagnosis, who were blinded to all clinical and prognostic information. The delineation ensured complete coverage of the plaque while avoiding the vessel lumen and surrounding normal tissue. All segmentation results were subsequently reviewed and validated by a senior radiologist with a senior professional title to ensure segmentation accuracy and consistency. To minimize segmentation bias, two radiologists delineated all ROIs by consensus before final validation by a senior radiologist; formal inter-observer reproducibility testing was subsequently performed on a random subset of 50 patients.

**Figure 2:**
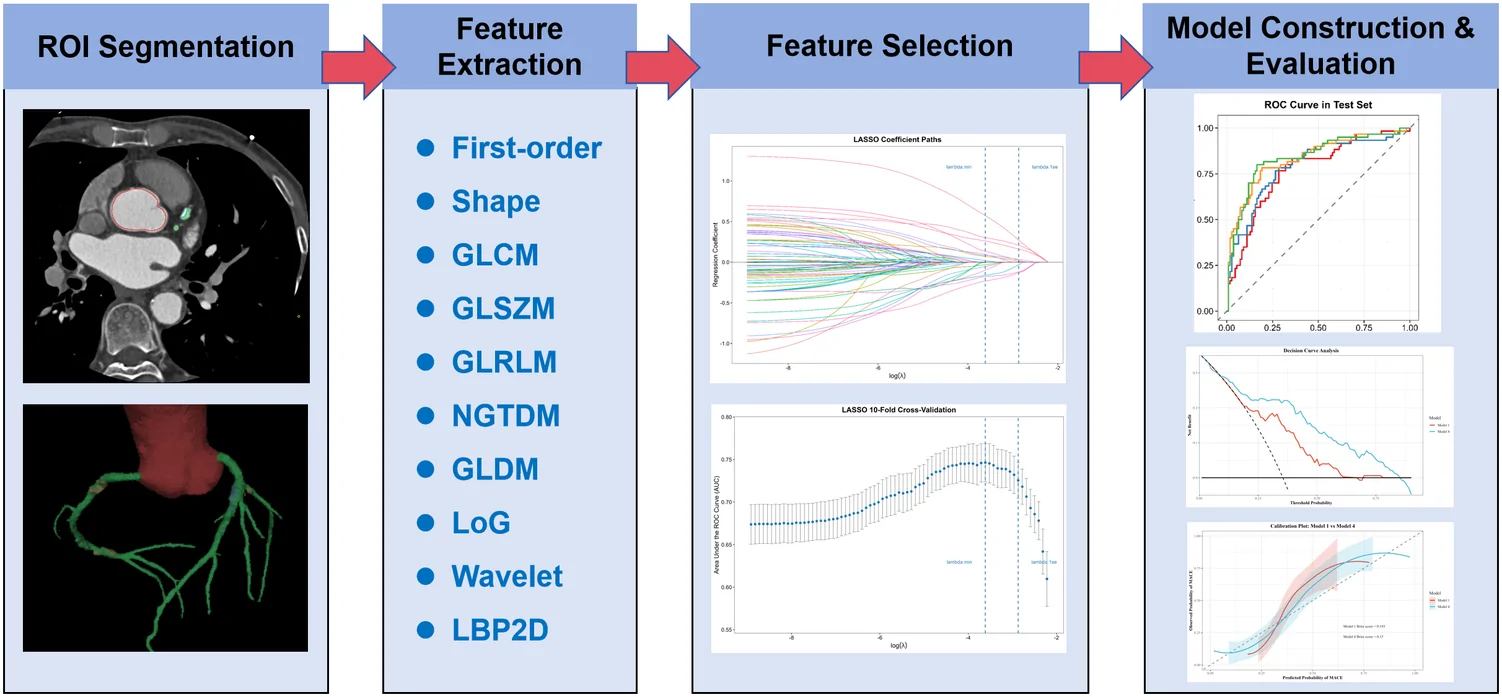
Flowchart of radiomic feature selection and predictive model construction. Note. Four core steps are shown from left to right: target coronary plaque ROI segmentation, radiomic feature extraction, feature selection, and predictive model construction and evaluation.

Plaque radiomic feature extraction was performed using the Neusoft Vasis Coronary CTA platform. Based on the delineated ROIs, radiomic features were extracted in strict accordance with the Image Biomarker Standardization Initiative (IBSI) guidelines, including morphological features, first-order grayscale statistical features, and multidimensional texture features. The texture features encompassed the gray-level co-occurrence matrix (GLCM), gray-level run length matrix (GLRLM), gray-level size zone matrix (GLSZM), gray-level dependence matrix (GLDM), and neighborhood gray-tone difference matrix (NGTDM). Following image filtering using Laplacian of Gaussian (LoG) and wavelet transform, a total of 1,833 quantitative radiomic features were ultimately extracted. To avoid overfitting, a hierarchical dimensionality reduction strategy was implemented, with all selection steps performed exclusively within the training set; the test set was entirely excluded from the feature selection process. First, variance filtering was applied. Subsequently, Z-score normalization was performed on the training set data, with the same parameters applied to the test set. After statistical testing (*t*-test or Mann–Whitney *U*-test, *P* < 0.05), the minimum redundancy maximum relevance (mRMR) algorithm was employed to retain 70 candidate features. Finally, 10-fold cross-validated least absolute shrinkage and selection operator (LASSO) regression was conducted, with lambda.1se selected as the optimal penalty parameter, yielding 9 core features with non-zero coefficients that were used to construct the radiomics score (RS). The complete workflow of radiomic feature selection and model construction is illustrated in Figure 2. To evaluate the inter-observer reproducibility of the selected radiomic features, 50 patients were randomly selected from the final cohort for independent, blinded re-segmentation by a second experienced radiologist. The intraclass correlation coefficient (ICC, two-way random-effects model, absolute agreement, single measures) was calculated for each of the 9 final features included in the radiomics model, with ICC > 0.75 predefined as the threshold for good reproducibility.

### 2.5 Predictive model construction

Univariable logistic regression analysis was performed on the clinical baseline characteristics and coronary plaque morphological features within the training set. A hybrid variable selection strategy was adopted: age, sex, hypertension, diabetes mellitus, hyperlipidemia, and smoking history were forced into the model as core clinical risk factors; the remaining clinical and plaque characteristics with *P* < 0.05 in the univariable analysis were also entered into the multivariable logistic regression model. Odds ratios (ORs) and 95% confidence intervals (CIs) were calculated for each variable to identify independent predictors of MACE. To determine the optimal modeling algorithm, three algorithms—elastic net logistic regression, XGBoost, and random forest—were compared within the training set. Hyperparameter optimization was performed via five-fold cross-validation and grid search. Elastic net logistic regression was ultimately selected to construct all predictive models, as this algorithm effectively handles high-dimensional features, corrects for multicollinearity among variables, and offers favorable stability and interpretability.

Based on the identified independent predictors and the radiomics score (RS), four predictive models were constructed: Model 1 (Clinical + Plaque), a conventional baseline model integrating clinical characteristics and plaque morphological features; Model 2 (Clinical + Radiomics), an exploratory model combining clinical characteristics and radiomic features; Model 3 (Plaque + Radiomics), an imaging-based model integrating plaque morphological and radiomic features; and Model 4 (All Features), a fully integrated model combining clinical, plaque, and radiomic features.

### 2.6 Statistical analysis and clinical utility

All statistical analyses were performed using R software (version 4.6.0). A two-sided *P* < 0.05 was considered statistically significant. Continuous variables following a normal distribution were expressed as mean ± standard deviation, and between-group comparisons were conducted using the independent samples *t*-test. Non-normally distributed continuous variables were expressed as median (interquartile range), and between-group comparisons were performed using the Mann–Whitney *U*-test. Categorical variables were expressed as frequency (percentage), and between-group comparisons were assessed using the χ^2^-test or Fisher's exact test, as appropriate.

Continuous variables were standardized using Z-score normalization. The normalization parameters (mean and standard deviation) were estimated exclusively from the training set; these parameters were then directly applied to standardize the test set without recalculation. Model discrimination was evaluated using the area under the receiver operating characteristic (ROC) curve (AUC). Harrell's C-index was further calculated as a sensitivity analysis to assess model discrimination for time-to-event MACE outcomes. The optimal cutoff threshold was determined by maximizing the Youden index in the training set and was subsequently applied directly to the test set for performance evaluation. The optimal threshold, accuracy, sensitivity, specificity, positive predictive value (PPV), negative predictive value (NPV), and Youden index were calculated for each model and validated in the test set. Model calibration was assessed using calibration curves and the Brier score. Since the primary objective of this study was to evaluate the incremental predictive value of radiomic features, a single prespecified primary comparison was defined: the difference in predictive performance between Model 1 (clinical + plaque baseline model) and Model 4 (combined full model). The DeLong test was used to compare AUCs between the two models, with a significance level of *α* = 0.05, and no correction for multiple comparisons was applied. The integrated discrimination improvement (IDI) and both continuous and categorical net reclassification improvement (NRI) were calculated to quantify the incremental predictive value of radiomics, with 95% confidence intervals estimated using 1,000 bootstrap resamples. Decision curve analysis (DCA) was performed to assess the net clinical benefit of the models. Model interpretability was assessed using the Kernel SHAP method, with the training set serving as the background dataset. The stability of feature importance was verified through 100 bootstrap resamples, and the independent contribution of each radiomic feature to the outcome was quantified. Tube voltage sensitivity analysis was performed by excluding all 100 kV scans to verify the robustness of the results, with the feature set and modeling pipeline unchanged. A sensitivity analysis comparing the 6-week and 6-month revascularization thresholds was also conducted to evaluate the robustness of the findings to the definition of the revascularization time window. Full results of all sensitivity analyses and C-index comparisons are provided in Supplementary Tables S3–S5.

## 3 Results

### 3.1 Baseline characteristics of the study population

A total of 567 patients with CAD were ultimately included in this study, among whom 202 experienced MACE during follow-up and 365 did not. Patients were allocated to a training set (*n* = 398) and a test set (*n* = 169) using stratified random sampling at a 7:3 ratio. The baseline characteristics of the two groups are summarized in Table 1. Age, the prevalence of hypertension and hyperlipidemia, and plaque volume, stenosis severity, plaque burden, and the prevalence of vulnerable plaques were significantly higher in the MACE group than in the non-MACE group (all *P* < 0.05). No statistically significant differences were observed in any baseline characteristics between the training and test sets (all *P* > 0.05, Table 2), indicating balanced dataset partitioning without selection bias.

**Table 1:** Baseline characteristics of the study population.

**Category**	**Variables**	**Total Cohort**	**Non-MACE**	**MACE**	***P* Value**
Demographic Characteristics	Age (years)	63 (57–68)	63 (57–67)	64 (58–70)	0.006
	Male Sex, *n* (%)	248 (43.7%)	161 (44.1%)	87 (43.1%)	0.880
Lifestyle Factors	Current Smoking, *n* (%)	87 (15.3%)	55 (15.1%)	32 (15.8%)	0.902
	Current Alcohol Drinking, *n* (%)	75 (13.2%)	49 (13.4%)	26 (12.9%)	0.955
Cardiovascular Risk Factors	Hypertension, *n* (%)	258 (45.5%)	132 (36.2%)	126 (62.4%)	<0.001
	Diabetes Mellitus, *n* (%)	154 (27.2%)	96 (26.3%)	58 (28.7%)	0.603
	Hyperlipidemia, *n* (%)	272 (48%)	160 (43.8%)	112 (55.4%)	0.010
	Total Cholesterol (mmol/L)	4.96 (4.37–5.57)	4.93 (4.37–5.49)	4.98 (4.46–5.65)	0.126
	Triglyceride (mmol/L)	1.66 (1.25–2.09)	1.66 (1.23–2.07)	1.68 (1.31–2.11)	0.261
	HDL-Cholesterol (mmol/L)	1.15 (1.06–1.37)	1.16 (1.07–1.37)	1.15 (1.04–1.41)	0.618
	LDL-Cholesterol (mmol/L)	3.03 (2.39–3.65)	3.02 (2.41–3.72)	3.05 (2.36–3.59)	0.634
	C-Reactive Protein (mg/L)	3.3 (1.55–5.3)	2.9 (1.29–4.7)	4.45 (2.26–8.18)	<0.001
	Fasting Glucose (mmol/L)	5.58 (5.01–6.24)	5.56 (5.01–6.19)	5.59 (5.01–6.36)	0.141
Plaque Imaging Characteristics	Remodeling Index	1.2 (1.01–1.61)	1.12 (1.01–1.53)	1.35 (1.04–1.69)	<0.001
	Stenosis Severity (%)	49 (40–60)	42 (40–60)	60 (40–70)	<0.001
	Plaque Burden (%)	11.61 (5.82–18.7)	9.82 (5.24–15.36)	17 (9.19–26.99)	<0.001
	Vulnerable Plaque, *n* (%)	220 (38.8%)	100 (27.4%)	120 (59.4%)	<0.001
	Overall, n	567	365	202	

Note. Continuous data are expressed as mean ± standard deviation for normally distributed variables or median (interquartile range, IQR) for non-normally distributed variables. Categorical data are expressed as *n* (%), where percentages are within-group percentages. *P* values were calculated using independent *t*-test, Mann–Whitney *U*-test, *χ*^2^-test, or Fisher's exact test as appropriate. *P* < 0.05 was considered statistically significant. Vulnerable plaque was defined as ≥2 high-risk features on CCTA: positive remodeling, low-attenuation plaque, spot calcification, napkin-ring sign. Total cohort *N* = 567; Non-MACE *N* = 365; MACE *N* = 202.

**Table 2:** Comparison of baseline characteristics between training Set and test Set.

Characteristic Category	Variable	Training Set *N* = 398	Test Set *N* = 169	*P* Value
Demographic Characteristics	Age (years)	63 (57–68)	63 (57–69)	0.774
	Male Sex, *n* (%)	180 (45.2%)	68 (40.2%)	0.316
Lifestyle Factors	Current Smoking, *n* (%)	63 (15.8%)	24 (14.2%)	0.715
	Current Alcohol Drinking, *n* (%)	56 (14.1%)	19 (11.2%)	0.439
Cardiovascular Risk Factors	Hypertension, *n* (%)	186 (46.7%)	72 (42.6%)	0.417
	Diabetes mellitus, *n* (%)	116 (29.1%)	38 (22.5%)	0.127
	Hyperlipidemia, *n* (%)	193 (48.5%)	79 (46.7%)	0.773
	Total Cholesterol (mmol/L)	4.97 (4.37–5.56)	4.93 (4.43–5.66)	0.568
	Triglyceride (mmol/L)	1.67 (1.24–2.11)	1.65 (1.31–2.04)	0.993
	HDL-Cholesterol (mmol/L)	1.15 (1.06–1.37)	1.17 (1.06–1.38)	0.527
	LDL-Cholesterol (mmol/L)	3.02 (2.36–3.64)	3.09 (2.41–3.69)	0.401
	C-Reactive Protein (mg/L)	3.37 (1.56–5.49)	3.3 (1.32–4.8)	0.275
	Fasting Glucose (mmol/L)	5.6 (5.05–6.25)	5.49 (4.95–6.15)	0.255
Plaque Imaging Characteristics	Remodeling Index	1.2 (1.02–1.61)	1.2 (1.01–1.61)	0.505
	Stenosis Severity (%)	48 (40–60)	55 (40–65)	0.147
	Plaque Burden (%)	11.35 (5.62–19.14)	12.29 (6.22–17.37)	0.961
	Vulnerable Plaque, *n* (%)	158 (39.7%)	62 (36.7%)	0.563
Outcome	MACE, *n* (%)	142 (35.7%)	60 (35.5%)	1

Note. Data are presented as mean ± standard deviation for normally distributed continuous variables, median [interquartile range (IQR)] for non-normally distributed continuous variables, and *n* (%) for categorical variables. *P* values were calculated using independent *t*-test or Mann–Whitney *U*-test for continuous variables, and Chi-square test or Fisher's exact test for categorical variables.

### 3.2 Identification of independent predictors

**Figure 3:**
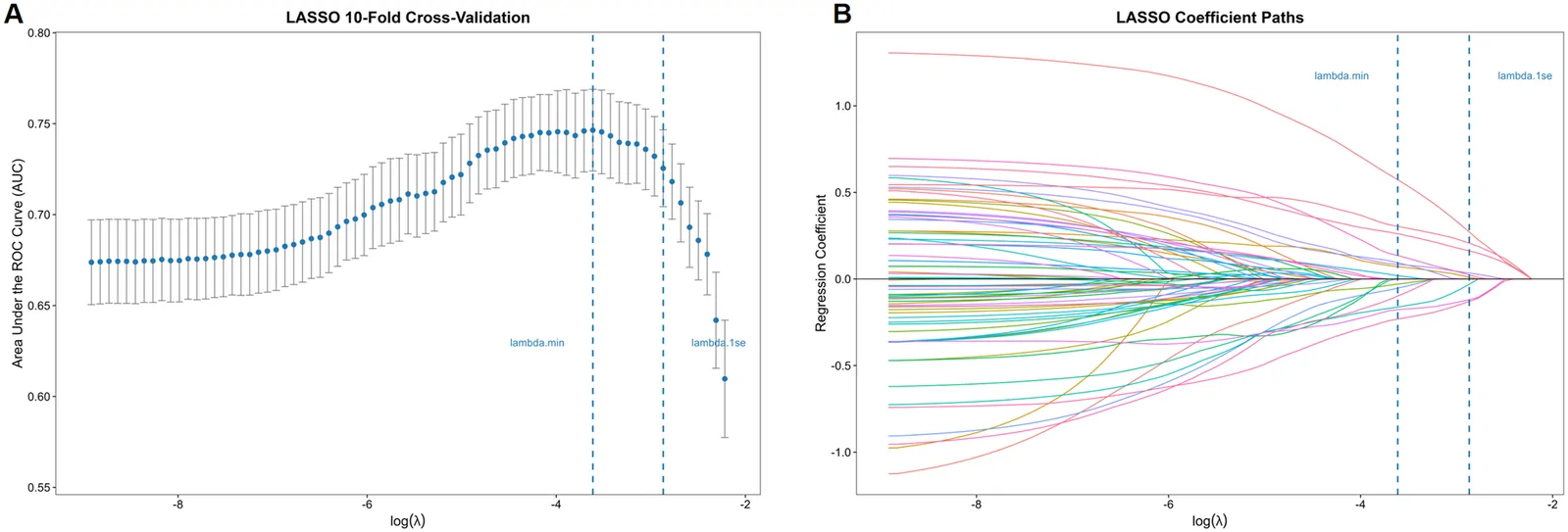
Results of radiomic feature selection based on LASSO regression. **(A)** Optimal lambda selection via 10-fold cross-validation of LASSO regression. The vertical dashed line indicates the optimal lambda value (lambda.min). **(B)** Coefficient paths of radiomic features in LASSO regression, showing the shrinkage process of regression coefficients as lambda changes.

Based on the training set data, a hierarchical dimensionality reduction strategy was employed, ultimately identifying 9 core radiomic features with non-zero regression coefficients to construct the radiomics score (RS). The cross-validation process and coefficient shrinkage paths for feature selection are illustrated in Figures 3A,B. These 9 features comprised 3 first-order grayscale statistical features, 2 gray-level co-occurrence matrix (GLCM) texture features, 2 gray-level run-length matrix (GLRLM) features, and 2 gray-level size zone matrix (GLSZM) features, thereby characterizing plaque microstructural properties from multiple dimensions, including grayscale distribution and spatial textural heterogeneity. The selected features and their corresponding regression coefficients are presented in Supplementary Table S1. In the inter-observer reproducibility analysis, 8 of the 9 final radiomic features showed ICC values >0.75, indicating good delineation stability. One wavelet-based texture feature presented an ICC of 0.697, slightly below the predefined threshold. The full ICC values with corresponding 95% confidence intervals are summarized in Supplementary Table S2.

For clinical and plaque morphological characteristics, a hybrid variable selection strategy was adopted to identify independent predictors. Age, sex, smoking history, hypertension, diabetes mellitus, and hyperlipidemia were forced into the model as core clinical risk factors, while the remaining variables were screened by univariable logistic regression. Variables with *P* < 0.05 in the univariable analysis were subsequently entered into the multivariable logistic regression model. Univariable logistic regression analysis demonstrated that hypertension, hyperlipidemia, triglycerides, stenosis severity, vulnerable plaques, and plaque burden were significantly associated with the risk of MACE (all *P* < 0.05) (Table 3). Notably, although C-reactive protein exhibited a significant between-group difference in the baseline characteristic analysis, it did not demonstrate predictive value in the univariable logistic regression and was therefore not included in the multivariable analysis.

**Table 3:** Univariate logistic regression analysis for MACE.

**Variable**	**OR (95% CI)**	***P* Value**
Age	1.23 (1–1.52)	0.056
Sex	1.04 (0.68–1.56)	0.87
Current Smoking	1.13 (0.64–1.96)	0.663
Current Alcohol Drinking	1.1 (0.6–1.95)	0.759
Hypertension	3.49 (2.28–5.41)	<0.001
Diabetes Mellitus	1.41 (0.9–2.2)	0.129
Hyperlipidemia	1.87 (1.23–2.84)	0.003
Total Cholesterol	1.22 (0.99–1.51)	0.058
Triglyceride	1.29 (1.05–1.61)	0.019
HDL-Cholesterol	1.02 (0.83–1.25)	0.823
LDL-Cholesterol	0.63 (0.07–1.12)	0.687
C-Reactive Protein	1.08 (0.88–1.34)	0.463
Fasting Glucose	1.07 (0.87–1.31)	0.513
Remodeling Index	1.14 (0.93–1.45)	0.216
Stenosis Severity	1.46 (1.19–1.81)	<0.001
Vulnerable Plaque	3.92 (2.56–6.08)	<0.001
Plaque Burden	2.32 (1.84–2.98)	<0.001

Note. Univariate logistic regression analysis of associations between clinical and imaging variables and MACE. OR, odds ratio; CI, confidence interval; MACE, major adverse cardiovascular events.

The results of the multivariable logistic regression analysis are presented in Table 4. Ultimately, hypertension (OR = 3.09, 95% CI: 1.90–5.09, *P* < 0.001), hyperlipidemia (OR = 1.96, 95% CI: 1.16–3.33, *P* = 0.013), vulnerable plaques (OR = 3.81, 95% CI: 2.32–6.35, *P* < 0.001), and plaque burden (OR = 2.27, 95% CI: 1.70–3.09, *P* < 0.001) emerged as independent risk factors for MACE. Among these, vulnerable plaques and plaque burden demonstrated the strongest predictive effects, while the remaining clinical and lipid-related parameters did not attain independent statistical significance. Based on these independent predictors, the clinical-plaque conventional baseline model (Model 1) was constructed.

**Table 4:** Multivariable logistic regression for MACE predictors.

**Variable**	**OR (95% CI)**	***P* Value**
Age	1.18 (0.92–1.52)	0.207
Sex	0.89 (0.5–1.59)	0.701
Current Smoking	0.92 (0.43–1.93)	0.818
Hypertension	3.09 (1.9–5.09)	<0.001
Diabetes Mellitus	0.98 (0.56–1.69)	0.944
Hyperlipidemia	1.96 (1.16–3.33)	0.013
Triglyceride	1.09 (0.85–1.4)	0.473
Stenosis Severity	0.98 (0.74–1.31)	0.907
Vulnerable Plaque	3.81 (2.32–6.35)	<0.001
Plaque Burden	2.27 (1.7–3.09)	<0.001

Multivariable logistic regression was performed using a hybrid variable selection strategy: core clinical risk factors were forced into the model, while other variables with *P* < 0.05 in univariate analysis were included. OR, odds ratio; CI, confidence interval; MACE, major adverse cardiovascular events.

### 3.3 Discriminative performance of predictive models

**Figure 4:**
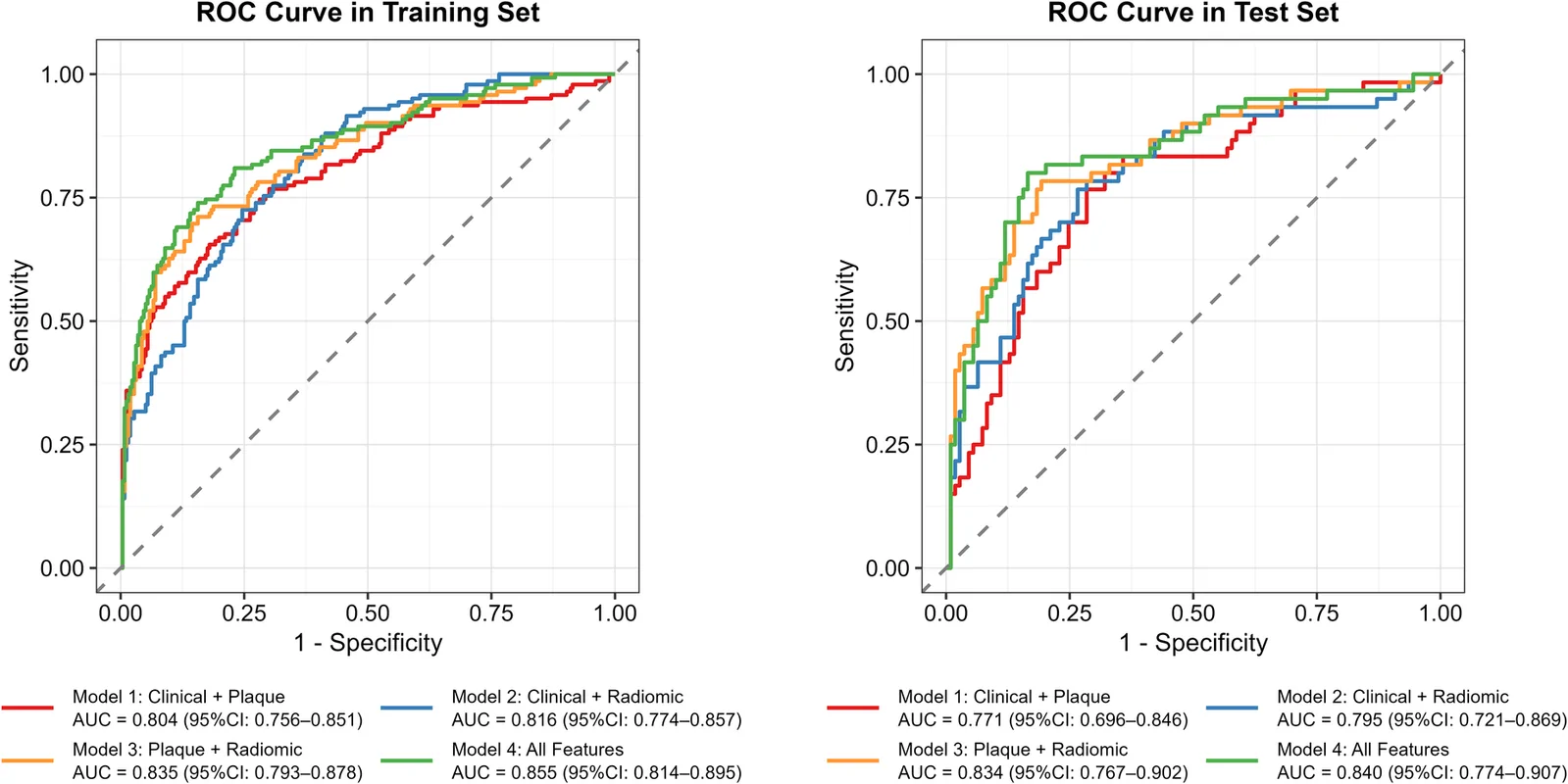
ROC curves of four predictive models for MACE in the training set and test set. Model 1: Clinical + Plaque baseline model; Model 2: Clinical + Radiomics model; Model 3: Plaque + Radiomics model; Model 4: Combined Clinical + Plaque + Radiomics model. AUC, area under the curve.

The ROC curves and AUC values of the four predictive models constructed based on the training set are presented for both the training and test sets in Figure 4, and the discrimination and performance metrics of each model in the test set are summarized in Table 5. In the test set, Model 1 (clinical + plaque) achieved an AUC of 0.771 (95% CI: 0.696–0.846), Model 2 (clinical + radiomics) achieved an AUC of 0.795 (95% CI: 0.721–0.869), Model 3 (plaque + radiomics) achieved an AUC of 0.834 (95% CI: 0.767–0.902), and Model 4 (all features) yielded the highest AUC of 0.840 (95% CI: 0.774–0.907), indicating optimal discriminative performance for MACE. Further analysis revealed that Model 4 also attained the highest accuracy (0.822), specificity (0.835), positive predictive value (0.727), and Youden index (0.635) among the four models, along with the lowest Brier score (0.150), suggesting superior concordance between predicted probabilities and observed outcomes. In addition, Model 4 achieved the highest C-index of 0.733 (95% CI: 0.653–0.812) in the test set (Table 5). Full C-index results for both the training and test cohorts are provided in Supplementary Table S3. Tube voltage sensitivity analysis in the 120 kV-only subset showed stable model performance and unchanged model ranking (Supplementary Table S4), confirming the robustness of the conclusions. A sensitivity analysis using an alternative 6-month threshold for revascularization events yielded consistent model performance and unchanged model ranking (Supplementary Table S5), confirming that our conclusions were robust to the definition of the revascularization time window.

**Table 5:** Performance of MACE prediction models in the test Set.

**Prediction Model**	**Optimal Threshold**	**Accuracy**	**Sensitivity**	**Specificity**	**PPV**	**NPV**	**Youden Index**	**Brier Score**	**AUC (95% CI)**	**C-index (95% CI)**
Model 1 (Clinical + Plaque)	0.346	0.734	0.767	0.716	0.597	0.848	0.482	0.193	0.771 (0.696–0.846)	0.678 (0.599–0.757)
Model 2 (Clinical + Radiomics)	0.335	0.746	0.767	0.734	0.613	0.851	0.501	0.171	0.795 (0.721–0.869)	0.711 (0.632–0.791)
Model 3 (Radiomics + Plaque)	0.349	0.799	0.783	0.807	0.691	0.871	0.591	0.157	0.834 (0.767–0.902)	0.724 (0.645–0.803)
Model 4 (All Features)	0.375	0.822	0.800	0.835	0.727	0.883	0.635	0.150	0.840 (0.774–0.907)	0.733 (0.653–0.812)

AUC, area under the receiver operating characteristic curve; CI, confidence interval; PPV, positive predictive value; NPV, negative predictive value. C-index, Harrell's concordance index for time-to-event MACE accounting for follow-up time and censoring. All 95% confidence intervals were calculated via 1,000 bootstrap resamples.

Comparison across the four models demonstrated that the incorporation of radiomic features substantially improved model discrimination relative to the clinical-plaque baseline model alone, and that the combined model integrating all clinical, plaque, and radiomic features achieved the optimal predictive performance.

### 3.4 Incremental predictive value of radiomics

The incremental predictive value of Model 4 (all features) was validated against Model 1 (clinical + plaque), with results presented in Table 6. The DeLong test demonstrated that the AUC of Model 4 was significantly higher than that of Model 1 (*P* = 0.016), indicating a statistically significant improvement in model discrimination following the incorporation of radiomic features. IDI analysis revealed that Model 4 achieved an IDI of 0.203 (95% CI: 0.151–0.256), suggesting that the overall discriminative capacity for MACE risk was significantly enhanced relative to the baseline model.

**Table 6:** AUC comparison and incremental predictive performance.

**Model Comparison**	**DeLong *P*-value**	**IDI (95% Bootstrap CI)**	**Categorical NRI (95% Bootstrap CI)**	**Continuous NRI (95% Bootstrap CI)**
Model 1 vs. Model 4	0.016	0.203 (0.151–0.256)	0.379 (0.245–0.508)	1.151 (0.88–1.416)

Categorical NRI was calculated using established cardiovascular risk thresholds: <5%, 5%–20%, >20%. Bootstrap 95% CIs were derived with 1,000 resamples. Model 1 vs. Model 4 was the prespecified primary comparison.

The categorical NRI, calculated based on prespecified cardiovascular risk stratification thresholds, was 0.379 (95% CI: 0.245–0.508), indicating that Model 4 more accurately reclassified patients into the appropriate risk categories. The continuous NRI, which is independent of prespecified thresholds, was 1.151 (95% CI: 0.88–1.416). The continuous NRI quantifies the overall improvement in risk ranking across the entire patient population; as a metric without a theoretical upper bound, higher values indicate more pronounced enhancement in risk discrimination capacity. These findings suggest that radiomic features not only improve patient reclassification under prespecified thresholds but also optimize risk ranking across the full risk spectrum. This substantial improvement is attributable to the ability of radiomics to capture plaque microheterogeneity that conventional methods cannot adequately reflect. Moreover, these results were mutually corroborated by the IDI and DeLong test results, further supporting the robustness of the conclusions.

### 3.5 Model calibration and clinical Net benefit

**Figure 5:**
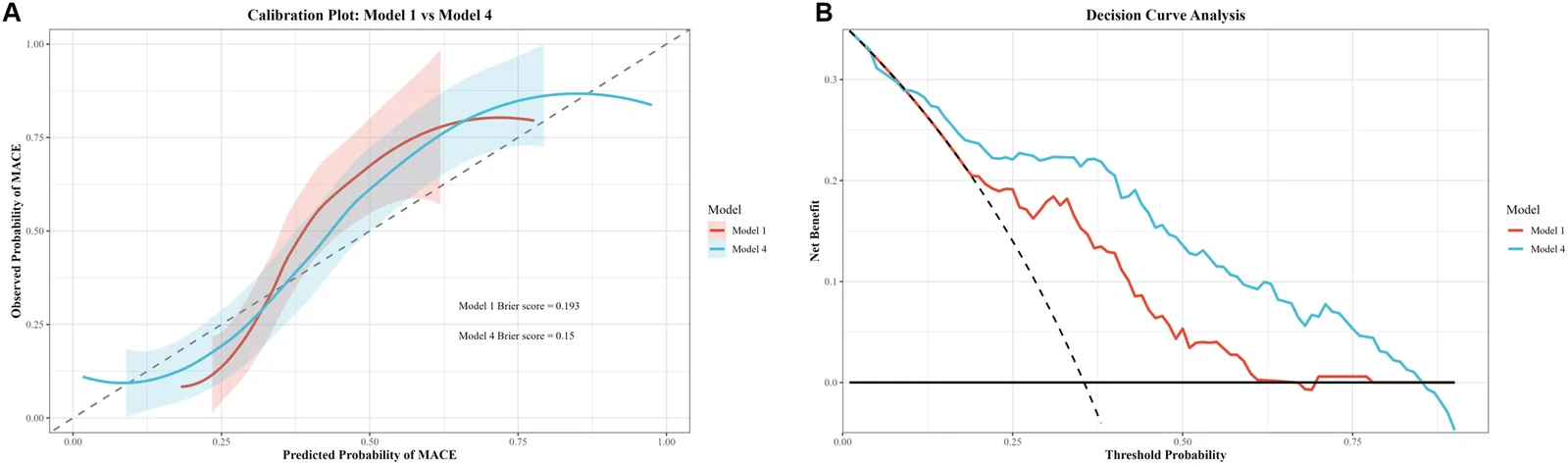
Calibration curves and decision curve analysis of model 1 and model 4. **(A)** Calibration curves, where the dashed line represents ideal prediction; **(B)** Decision curve analysis, with net benefit on the *y*-axis. Model 1: Clinical + plaque features; Model 4: Combined full model.

The calibration curves and decision curve analysis (DCA) for Model 1 and Model 4 are presented in Figure 5. The calibration curves demonstrated favorable concordance between the predicted probabilities and the observed MACE rates for Model 4. Moreover, Model 4 yielded a substantially lower Brier score (0.150) compared with Model 1 (0.193), indicating that the full-feature combined model provided more reliable probability estimates. Decision curve analysis showed that Model 4 consistently achieved a higher net benefit than the baseline Model 1 across a clinically relevant range of risk thresholds, suggesting that the incorporation of radiomic features confers superior clinical decision-making value for MACE risk stratification in patients with CAD.

### 3.6 Interpretability of radiomic features via SHAP analysis

**Figure 6:**
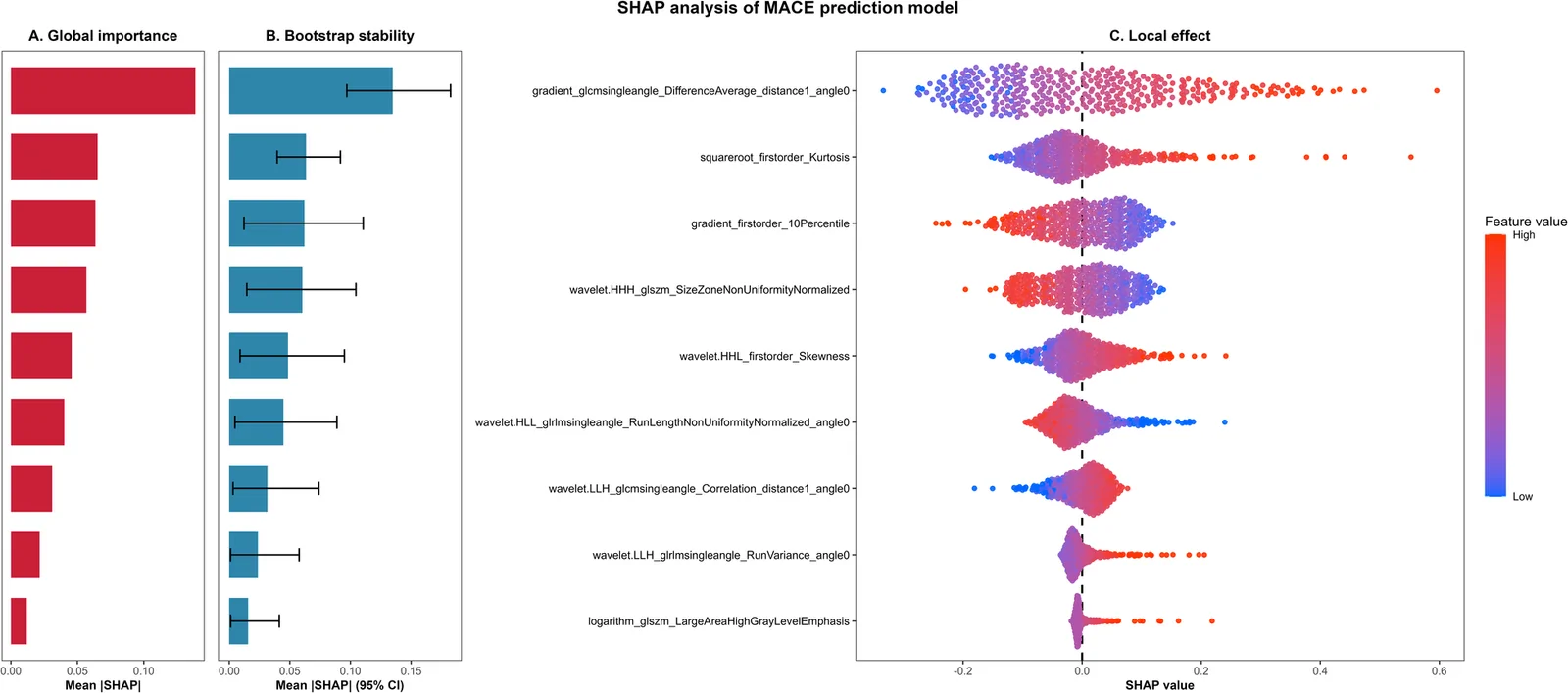
SHAP analysis of radiomic features in the MACE prediction model. **(A)** Global feature importance ranking based on mean absolute SHAP values;**(B)** Bootstrap stability of feature importance with 95% confidence intervals;**(C)** Local feature effects on MACE risk presented as a SHAP summary swarm plot.

The interpretability of the optimal predictive model was assessed using the SHapley Additive exPlanations (SHAP) method. The Kernel SHAP algorithm was employed, with the training set serving as the background dataset, and 100 bootstrap resamples were performed to evaluate the stability of feature importance and to calculate 95% confidence intervals (Figure 6). Global feature importance ranking based on mean absolute SHAP values revealed that gray-level co-occurrence matrix (GLCM) texture features were the primary drivers of MACE prediction, with substantially higher contribution than other features. The remaining core features encompassed first-order statistical features and wavelet-domain texture features, which comprehensively reflect plaque grayscale distribution, spatial heterogeneity, and structural complexity. The SHAP summary plot demonstrated that higher values of core texture features were positively associated with increased MACE risk, whereas higher values of certain first-order statistical features were associated with reduced MACE risk, indicating that different radiomic features exert distinct directional regulatory effects on risk prediction. The bootstrap resampling results showed that the 95% confidence intervals of the core feature importance were narrow, confirming that the feature importance assessments were stable and reliable. These findings substantiate that texture features reflecting plaque microheterogeneity serve as core imaging markers for MACE prediction, providing a reliable interpretability basis for the clinical decision-making value of the model.

## 4 Discussion

Clinicians lack accurate tools for individualized MACE risk stratification among CAD patients. To address this need, we constructed and validated a multi-dimensional machine learning model combining clinical data, routine plaque metrics, and CCTA plaque radiomics. Our core findings can be summarized as follows: First, the fully integrated model combining all multidimensional features (Model 4) achieved optimal predictive performance in the independent test set, with an AUC of 0.840, significantly outperforming the conventional baseline model comprising only clinical and plaque features (Model 1, AUC = 0.771, *P* = 0.016). Second, radiomic features conferred significant and independent incremental predictive value beyond the conventional model, with an IDI of 0.203 and a categorical NRI of 0.379, demonstrating that the incorporation of radiomics substantially improved patient risk stratification and reclassification capacity. Third, SHAP-based interpretability analysis revealed that texture features reflecting plaque micro-grayscale heterogeneity were the core drivers of MACE prediction, providing a robust pathophysiological basis for the clinical translation of the model.

In recent years, multiple studies have constructed MACE risk prediction models for CAD patients based on CCTA plaque radiomics, with the core focus consistently directed toward the incremental predictive value of radiomics. Secondary analyses of the large-scale clinical trials SCOT-HEART and PROMISE have established that conventional high-risk plaque features are independent predictors of future cardiac events (17, 18). However, as underscored by a 2023 systematic review and meta-analysis published in JACC: Cardiovascular Imaging, which encompassed 30 studies and over 30,000 subjects, although traditional high-risk plaque imaging features such as thin-cap fibroatheroma (TCFA) and low-attenuation plaque are statistically significantly associated with MACE, their positive predictive value remains generally low, substantially limiting the utility of these markers alone for clinical risk stratification (19, 20). To address this clinical challenge, plaque radiomics has emerged as a novel technical pathway for the non-invasive identification of vulnerable plaques and prognostic risk stratification. Kolossváry et al. (21), based on the SCOT-HEART study, demonstrated that plaque radiomics independently predicted fatal and non-fatal myocardial infarction, with long-term prognostic predictive value significantly superior to conventional plaque burden metrics, effectively capturing plaque texture and spatial heterogeneity and providing independent incremental prognostic information. Lin et al. (22) reported that a model integrating radiomic and conventional plaque features achieved an AUC of 0.86 for discriminating culprit plaques—lesions retrospectively identified as the source of acute coronary events—significantly outperforming the conventional plaque-only model. Notably, their study relied on *post-hoc* lesion identification after clinical events, whereas our analysis adopted a standardized, outcome-blind target plaque selection protocol applied uniformly to all patients at baseline. Chen et al. (23) constructed a CCTA-based vulnerable plaque radiomic signature using intravascular ultrasound as the reference standard and confirmed it as an independent predictor of MACE in CAD patients, thereby establishing a pathophysiological foundation for plaque radiomics research. However, these studies predominantly adopted an analytical strategy of indirectly deriving prognostic information from diagnostic labels, lacking end-to-end direct modeling targeting the clinical hard endpoint of MACE. In the present study, we directly constructed a multidimensional combined model with MACE as the endpoint. The results demonstrated that the full-variable model achieved an AUC of 0.840, representing a significant improvement over the conventional model, with IDI and NRI further confirming the independent incremental value of radiomics, thereby providing a more precise tool for clinical non-invasive risk stratification. The epidemiological observation that approximately 70% of acute cardiovascular events originate from non-obstructive plaques is derived from population-based studies in which non-obstructive lesions vastly outnumber obstructive ones. Our cohort consisted exclusively of patients with established CAD confirmed by CCTA, who inherently harbor a greater burden of obstructive disease. Nevertheless, the lower quartile of stenosis severity in the MACE group was 40%, indicating that at least one-quarter of events in our cohort occurred in patients with non-obstructive plaques. This finding is consistent with the prognostic relevance of non-obstructive vulnerable plaques and further underscores the necessity of risk stratification beyond stenosis severity alone.

An important innovation of this study lies in our employment of multiple statistical methods to comprehensively quantify the incremental predictive value of radiomic features, rather than relying solely on AUC comparison. Unlike AUC, IDI and NRI more directly reflect the degree of improvement in patient risk stratification conferred by the model, bearing clearer clinical significance. Our results demonstrated that, following the addition of radiomic features to the conventional clinical-plaque model, the IDI reached 0.203, indicating that the overall discriminative capacity for MACE risk was enhanced by 20.3%; the categorical NRI reached 0.379, signifying that approximately 37.9% of patients were more accurately reclassified into the appropriate risk categories. This finding carries important clinical implications: modest improvements in AUC may not necessarily translate into clinical benefit, whereas NRI and IDI directly reflect the potential impact of the model on patient management decisions. Our results robustly demonstrate that radiomic features do not merely recapitulate conventional indicators but rather provide independent, clinically valuable incremental prognostic information capable of substantially enhancing the risk prediction capacity of conventional models.

The SHAP-based interpretability analysis conducted in this study further elucidated the intrinsic predictive logic of the model. Global importance ranking revealed that the predictive performance of the model was primarily driven by texture features reflecting plaque microstructural heterogeneity, among which the gray-level co-occurrence matrix feature gradient_glcmsingleangle_DifferenceAverage_distance1_angle0 exhibited the highest contribution. This finding is highly concordant with the pathophysiological mechanisms of atherosclerosis: the heterogeneity of grayscale distribution within plaques essentially reflects the uneven spatial distribution of lipid cores, fibrous tissue, and calcified components, and such compositional inhomogeneity constitutes a core pathological hallmark of vulnerable plaques (24). When a large lipid-rich necrotic core and a thin fibrous cap are present within a plaque, the heterogeneity of grayscale distribution increases substantially, leading to an elevated value of this feature, which is in turn associated with increased MACE risk. Furthermore, we observed that the first-order statistical feature gradient_firstorder_10Percentile was negatively correlated with MACE risk, a finding consistent with clinical understanding: elevated values of this feature indicate increased overall plaque density and a higher degree of calcification, and calcified plaques are generally more stable with a lower risk of rupture. These results not only provide interpretability support for the clinical translation of the model but also delineate a clear direction for the subsequent development of more targeted imaging biomarkers. Notably, C-reactive protein demonstrated a significant between-group difference at baseline (4.45 vs. 2.9 mg/L, *P* < 0.001) but did not retain predictive value in univariable logistic regression (OR = 1.08, *P* > 0.05). This discrepancy is likely attributable to the highly right-skewed distribution of CRP, which may attenuate its statistical association with MACE when modeled on the original scale. Furthermore, the inflammatory signal captured by CRP may be partially mediated or superseded by imaging variables such as plaque burden and vulnerable plaque status, which directly reflect the cumulative structural consequences of inflammatory processes. Future studies may consider log-transformation or non-linear modeling approaches to more comprehensively evaluate the independent predictive contribution of circulating inflammatory biomarkers within radiomics-based models.

Beyond plaque-based radiomics, pericoronary adipose tissue (PCAT) serves as a key regulatory target of coronary vascular wall inflammation: vascular wall inflammation can inhibit lipid accumulation in PCAT preadipocytes through paracrine signaling, leading to alterations in the composition, structure, and imaging characteristics of PCAT, which can be precisely quantified via CCTA radiomics (25). Oikonomou et al. (13) constructed a perivascular adipose tissue radiotranscriptomic signature (FRP) based on CCTA, which significantly enhanced MACE prediction in a multicenter prospective cohort, achieving a C-index of 0.88 when combined with traditional risk factors. Lin et al. (26) conducted a prospective case-control study demonstrating that PCAT radiomics effectively differentiated patients with acute myocardial infarction from those with stable CAD, with further improvement in discriminatory performance following the integration of clinical risk factors and PCAT attenuation values. The majority of current studies focus exclusively on plaque-based radiomics; however, plaque vulnerability is closely associated with local coronary inflammatory status, and PCAT radiomics can provide complementary information from the inflammatory dimension. Future integration of dual-region plaque and PCAT radiomics, simultaneously capturing the structural vulnerability of plaques and local coronary inflammatory status, holds promise for further improving MACE risk prediction performance.

Plaque vulnerability is a complex phenotype jointly determined by multiple factors including structural, biomechanical, and hemodynamic determinants, and single-dimensional radiomic features are insufficient to comprehensively decode its risk. Previous studies have demonstrated that biomechanical parameters such as wall shear stress and circumferential stress are closely associated with plaque progression and rupture risk, and that integrating radiomic and biomechanical parameters can significantly enhance the identification of vulnerable plaques and MACE prediction performance (27). Moreover, multimodal fusion modeling has emerged as an important direction in CAD risk assessment. Zou et al. (28) combined pericoronary adipose tissue radiomics with CT-derived fractional flow reserve (CT-FFR) and clinical imaging features, significantly improving MACE risk stratification capacity in hypertensive patients with CAD. Currently, most plaque radiomics studies rely on static image-based modeling, neglecting the impact of hemodynamic and mechanical factors on plaque vulnerability. Future studies may incorporate computational fluid dynamics simulations to derive biomechanical parameters of plaques, thereby constructing a “radiomics-mechanics” multimodal fusion model that comprehensively deciphers the biological nature of plaque vulnerability from structural, inflammatory, and functional dimensions.

The multidimensional predictive model centered on radiomics can serve as a non-invasive auxiliary tool, providing decision support for the individualized management of CAD patients. Its clinical application value is primarily manifested in three aspects: First, it enables precise risk stratification of CAD patients. For individuals identified as high-risk by the model, more aggressive secondary prevention strategies may be adopted, including intensified lipid-lowering therapy (e.g., PCSK9 inhibitors), colchicine targeted anti-inflammatory therapy, and shortened imaging and clinical follow-up intervals (29, 30); for low-risk individuals, unnecessary examinations and treatments may be avoided, thereby optimizing healthcare resource allocation. Second, it facilitates the early identification of vulnerable plaques by capturing micro-heterogeneity signals undetectable by conventional CCTA, thereby enabling prospective early warning of ACS. Third, it may serve as a non-invasive biomarker for evaluating the therapeutic efficacy of lipid-lowering, anti-inflammatory, and other pharmacological interventions, quantifying changes in plaque vulnerability through dynamic alterations in radiomic features before and after treatment, and guiding the adjustment of individualized therapeutic regimens.

This study has several limitations that warrant acknowledgment. First, this was a single-center retrospective study, which may be subject to selection bias; the generalizability of the model across different CT scanners, scanning parameters, and ethnic populations requires validation through multicenter prospective large-sample cohort studies. Second, plaque regions of interest were manually segmented. Although we minimized delineation bias through blinded independent verification by a senior radiologist and supplemented inter-observer reproducibility testing, a degree of inter-observer variability remains inherent to manual segmentation. In our reproducibility analysis, 8 of the 9 final radiomic features met the predefined ICC threshold of >0.75, while one wavelet-derived texture feature showed slightly lower reproducibility (ICC = 0.697), which is attributable to the inherent sensitivity of wavelet-transformed texture features to subtle differences in plaque boundary delineation. The elastic net regularization applied during model construction can further attenuate the influence of less stable features on overall performance, but cannot fully eliminate the variability introduced by manual segmentation. Future adoption of deep learning-based automated segmentation techniques is warranted to further improve reproducibility and clinical applicability (31). Third, this study included only coronary segments with a diameter >1.5 mm, potentially missing information on distal microvascular disease. Fourth, this study established only statistical associations between radiomic features and MACE, without integrating histopathological and molecular biological data to further elucidate the underlying biological mechanisms, which represents a core direction for subsequent research. Fifth, binary logistic regression was applied as the primary modeling framework given the complete 24-month follow-up and minimal censoring in this cohort, under which conditions binary endpoints provide intuitive prediction of 24-month MACE risk that aligns with clinical practice. Supplementary C-index analyses confirmed consistent model performance after incorporating temporal follow-up information, supporting the robustness of our conclusions. Future studies with extended follow-up periods should consider Cox proportional hazards regression or competing risk models as the primary analytical framework. Additionally, the MACE rate in this cohort was 35.6%, which reflects the inclusion of patients with established CAD rather than a general screening population. Consequently, the reported PPV and NPV are prevalence-dependent and may not be directly transportable to lower-risk populations. External validation in cohorts with diverse baseline risk profiles is warranted before clinical deployment.

In summary, our study demonstrates that CCTA plaque radiomics can decode microscopic plaque pathological heterogeneity that is imperceptible to conventional visual assessment, and its predictive value for MACE is independent of traditional clinical and plaque morphological features. The full-variable combined model integrating clinical risk factors, plaque morphology, and radiomic features significantly outperforms the conventional baseline model in terms of discrimination, calibration, and risk reclassification capacity, providing a novel technical pathway for the non-invasive quantitative assessment of coronary vulnerable plaques.

## 5 Conclusion

The multidimensional predictive model constructed in this study, integrating clinical, plaque morphological, and CCTA-derived radiomic features, enables accurate prediction of MACE risk in patients with coronary artery disease. This model can be implemented based on routine clinical CCTA examinations without the need for additional invasive procedures, and holds promise as an objective, reliable, non-invasive quantitative tool for individualized risk stratification, early intervention, and follow-up management of patients with CAD, thereby advancing precision risk stratification via routine non-invasive CCTA scans.

## Data Availability

The raw data supporting the conclusions of this article will be made available by the authors, without undue reservation.
