# Supplementary material for: A CCTA coronary plaque radiomic model for predicting major adverse cardiovascular events in patients with coronary artery disease
Source: Front Cardiovasc Med. 2026 Aug 20;13:1886018. doi: 10.3389/fcvm.2026.1886018 (PMC13539132; doi:10.3389/fcvm.2026.1886018)
Supplement: Supplementary file 1 [file Datasheet1.docx]

Supplementary Material

# 1 Supplementary Tables

| **Radiomic feature** | **Coefficient** |
| --- | --- |
| wavelet.LLH_glrlmsingleangle_RunVariance_angle0 | 0.00907053349589189 |
| gradient_firstorder_10Percentile | -0.030544235 |
| logarithm_glszm_LargeAreaHighGrayLevelEmphasis | 0.0345011676939259 |
| wavelet.HLL_glrlmsingleangle_RunLengthNonUniformityNormalized_angle0 | -0.119408533 |
| wavelet.LLH_glcmsingleangle_Correlation_distance1_angle0 | 0.0237220957642525 |
| wavelet.HHH_glszm_SizeZoneNonUniformityNormalized | -0.129446759 |
| wavelet.HHL_firstorder_Skewness | 0.162443320480153 |
| squareroot_firstorder_Kurtosis | 0.206970304662742 |
| gradient_glcmsingleangle_DifferenceAverage_distance1_angle0 | 0.271654759866904 |
| The radiomic signature (RadScore) was calculated as the linear combination of the nine selected features weighted by their respective LASSO coefficients. LASSO, least absolute shrinkage and selection operator. | |

## 1.1  Supplementary Table 1.The nine radiomic features selected by LASSO regression and their corresponding coefficients.

## 1.2  Supplementary Table 2. Inter-observer intraclass correlation coefficients (ICC) and 95% confidence intervals of the 9 final radiomic features.

| **Feature** | **ICC** | **95% CI** |
| --- | --- | --- |
| wavelet-LLH_glrlmsingleangle_RunVariance_angle0 | 0.864 | 0.771–0.92 |
| gradient_firstorder_10Percentile | 0.884 | 0.805–0.932 |
| logarithm_glszm_LargeAreaHighGrayLevelEmphasis | 0.779 | 0.639–0.868 |
| wavelet-HLL_glrlmsingleangle_RunLengthNonUniformityNormalized_angle0 | 0.697 | 0.521–0.816 |
| wavelet-LLH_glcmsingleangle_Correlation_distance1_angle0 | 0.844 | 0.74–0.908 |
| wavelet-HHH_glszm_SizeZoneNonUniformityNormalized | 0.76 | 0.611–0.857 |
| wavelet-HHL_firstorder_Skewness | 0.836 | 0.727–0.903 |
| squareroot_firstorder_Kurtosis | 0.863 | 0.771–0.92 |
| gradient_glcmsingleangle_DifferenceAverage_distance1_angle0 | 0.755 | 0.605–0.853 |
| Note: ICC, intraclass correlation coefficient (two-way random-effects model, absolute agreement, single measures); CI, confidence interval. | | |

## 1.3  Supplementary Table 3. Harrell’s C-index of four prediction models in training and test sets

| **Model** | **Dataset** | **Cindex** | **CI95** |
| --- | --- | --- | --- |
| Model 1（Clinical + Plaque） | Training set | 0.705 | 0.654-0.757 |
| Model 2（Clinical + Radiomics） | Training set | 0.716 | 0.664-0.767 |
| Model 3（Plaque + Radiomics） | Training set | 0.72 | 0.669-0.772 |
| Model 4（Combined Full） | Training set | 0.733 | 0.682-0.785 |
| Model 1（Clinical + Plaque） | Test set | 0.678 | 0.599-0.757 |
| Model 2（Clinical + Radiomics | Test set | 0.711 | 0.632-0.791 |
| Model 3（Plaque + Radiomics） | Test set | 0.724 | 0.645-0.803 |
| Model 4（Combined Full） | Test set | 0.733 | 0.653-0.812 |
| Note: C-index evaluates time-to-event discrimination; CI, confidence interval derived from 1000 bootstrap resamples. | | | |

## 1.4  Supplementary Table 4. Tube voltage sensitivity analysis.

| **Model** | **Train_AUC** | **Test_AUC** | **Train_Cindex** | **Test_Cindex** |
| --- | --- | --- | --- | --- |
| Model 1 | 0.7919 | 0.7627 | 0.7028 | 0.6708 |
| Model 2 | 0.8093 | 0.8024 | 0.7207 | 0.7217 |
| Model 3 | 0.8214 | 0.8409 | 0.7133 | 0.7185 |
| Model 4 | 0.8432 | 0.8448 | 0.731 | 0.73 |
| The 120 kV-only subset was obtained by excluding all 100 kV scans. AUC, area under the curve; C-index, concordance index. | | | | |

## 1.5  Supplementary Table 5. Sensitivity analysis comparing MACE definitions with 6-week versus 6-month revascularization thresholds in the test set.

| **Model** | **AUC_6-week** | **AUC_6-month** | **C-index_6-week** | **C-index_6-month** |
| --- | --- | --- | --- | --- |
| Model 1 | 0.77 (0.696-0.845) | 0.746 (0.653-0.838) | 0.678 (0.599-0.757) | 0.686 (0.592-0.781) |
| Model 2 | 0.796 (0.723-0.87) | 0.784 (0.688-0.88) | 0.711 (0.632-0.791) | 0.73 (0.635-0.825) |
| Model 3 | 0.833 (0.766-0.901) | 0.811 (0.718-0.904) | 0.724 (0.645-0.803) | 0.747 (0.652-0.842) |
| Model 4 | 0.841 (0.774-0.907) | 0.818 (0.725-0.911) | 0.733 (0.653-0.812) | 0.758 (0.663-0.853) |
| Note: AUC, area under the receiver operating characteristic curve; C-index, Harrell's concordance index. The 6-month threshold defined revascularization events occurring ≥ 6 months after the index CCTA examination. | | | | |
